# Adolescent Haze-Related Knowledge Level Study: A Cross-Sectional Survey With Sensitivity Analysis

**DOI:** 10.3389/fpubh.2020.00229

**Published:** 2020-07-09

**Authors:** Qingchun Zhao, Yuejia Zhao, Hongzhe Dou, Yanrong Lu, Yanhong Chen, Lingwei Tao

**Affiliations:** ^1^Affiliated Hospital of Hebei University, Baoding, China; ^2^United Front Department, Hebei University, Baoding, China; ^3^School of Public Health, Peking University, Beijing, China

**Keywords:** adolescent, haze, cognitive level, sensitivity analysis, health education

## Abstract

**Objective:** This study aimed to investigate the level of haze-related knowledge adolescents have and to explore relevant influencing factors.

**Methods:** From June 2015 to January 2016, researchers randomly selected 2 districts from the 20 districts of Baoding, China. Then, researchers randomly selected two middle schools from two districts. By conducting a stratified cluster sampling and considering one class as a unit, researchers randomly selected, from the other middle school, five first-grade classes, five second-grade classes, five third-grade classes from the one middle school, and three first-grade classes, two second-grade classes, and two third-grade classes. Finally, 1,100 adolescents were investigated by using the demographic questionnaire and the Adolescent Haze-Related Knowledge Awareness Assessment Scale (AHRKAAS). Multiple linear regressions were conducted to explore factors affecting the adolescent haze-related knowledge. Sensitivity analysis was used to confirm associations between influencing factors and AHRKAAS scores.

**Results:** The AHRKAAS score rate was 69.9%. The dimension of human factors of haze formation was the highest (score rate = 85.6%). The dimension of haze harms on the human body was the lowest (score rate = 57.1%). Compared with the group (monthly expenses <300 yuan), the group (monthly expenses ≥ 600 yuan) had a higher AHRKAAS score (β = 4.882, 95% CI: 0.979, 8.784). Compared with the group (Do not live with parents), the group (Live with parents) had a higher AHRKAAS score (β = 14.675, 95% CI: 9.494, 19.855). Compared with the group (Never undergo a physical examination), the group (Once a year) (β = 7.444, 95% CI: 2.922, 11.966) and the group (A few times a year) (β = 7.643, 95% CI: 2.367, 12.919) had a higher AHRKAAS score. Compared with the group (Know nothing), the group (Know most) (β = 9.623, 95% CI: 2.929, 16.316) and the group (Know very well) (β = 15.367, 95% CI: 7.220, 23.515) had a higher AHRKAAS score. These associations were still reliable and consistent in different sensitivity analysis models.

**Conclusion:** The level of adolescent haze-related knowledge is low and is affected by monthly expenses, living condition, physical examination frequency, and knowledge of respiratory system diseases. Government bodies, schools, and research institutions should strengthen cooperation of health publicity and health education to improve adolescent haze-related knowledge.

## Introduction

Air pollution has become an important global problem, affecting the health of millions of people around the world (1). Air pollution directly causes many serious public health issues. The Chinese Government started a National Plan on Air Pollution Control, which set out strict goals and measures to prevent and control air pollution in 2012 for the first time. In September 2013, the Chinese Government promulgated the first National Action Plan on Air Pollution Prevention and Control (2013–2017), which demands that the air quality will be significantly improved by 2017 (2). As a manifestation form of severe air pollution, haze has short-term acute harms and long-term chronic harms on human health. This can cause far-reaching impacts on body health, especially in the children and adolescents (3–8). Chen pointed out that children and adolescents, the elderly population, those with lower socioeconomic status (such as low educational background or low income level), and people with respiratory system diseases are more susceptible to haze (9). In order to understand the degree of haze-related awareness in the public, Liu conducted a survey of 330 residents in Chenzhou City, China. Liu's research results showed that 29.29% of residents increased the number of physical examinations to deal with the damage of haze to their health (10). Therefore, among these sensitive people, it is of importance to study their haze-related knowledge levels and possible influencing factors. Since adolescents are in a critical period of growth and development, adolescents' physical functions are not yet fully developed and thus more susceptible to the damaging effect of haze (1, 11–15). Acquiring knowledge related to haze can encourage adolescents to set up health protection awareness for preventing haze harms on body health. This should reduce the incidence of related diseases and allow for their normal growth and development. Furthermore, it can reduce the burden currently placed on the medical system, the government, schools, society, and even the patients' family (16). Therefore, our research team used the Adolescent Haze-Related Knowledge Awareness Assessment Scale (AHRKAAS) (17), developed in the early stage of our research, to conduct a cross-sectional survey and explore the related factors affecting the level of adolescent haze-related knowledge. In the aspect of adolescent health protection for haze, this study may provide an important theoretical framework for conducting behavioral interventions and health education among adolescents in the future.

## Methods

### Participants

**Figure 1:**
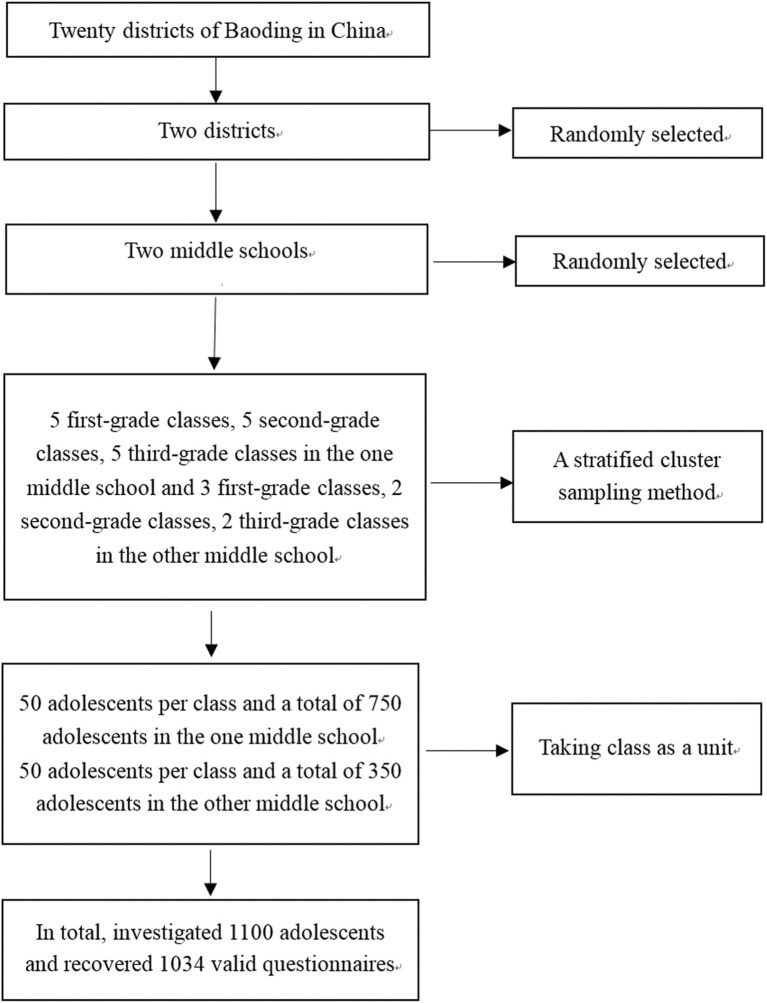
Study participant flow diagram.

From June 2015 to January 2016, in Baoding, China, our research team randomly selected 2 districts from 20 districts of Baoding. Subsequently, the research team randomly selected two middle schools from these two districts. Using a stratified cluster sampling method and by considering one class as a unit, the researchers randomly selected five first-grade classes, five second-grade classes, and five third-grade classes from the first middle school (50 adolescents per class, a total of 750 adolescents), and three first-grade classes, two second-grade classes, and two third-grade classes from the other middle school (50 adolescents per class, a total of 350 adolescents). In total, the research team investigated 1,100 adolescents from 22 classes between 11 and 20 years old (14.413 ± 1.597 years old). The inclusion criteria are as follows: (1) voluntarily participated in this research; (2) the adolescents who have an appropriate capacity to understand relevant content correctly and comprehend questionnaires as well as answer them; and (3) do not suffer from mental diseases, brain diseases, or other serious diseases. A total of 1,100 questionnaires were distributed; 1,034 valid questionnaires were returned. The valid recovery rate of questionnaires was 94%. Figure 1 represents the participant flow diagram.

### Survey Tools

Our survey tools included two parts—(1) Adolescent demographic characteristic questionnaire: The structured questionnaire was used to obtain the data pertaining to demographics, medical factors, and knowledge relating to respiratory disease (*know nothing* = 1, *know a little* = 2, *know a small part* = 3, *know most* = 4, *know very well* = 5). (2) AHRKAAS (Additional File 1): The AHRKAAS was developed by our research team in our earlier study [17). AHRKAAS Cronbach's α coefficient was 0.923; content validity was 0.940; criterion validity was 0.444 (regarding the score of the overall self-report item relating to haze-related knowledge as the criterion: “Assuming that the full score of haze-related knowledge is 100 points, how much do you think you are able to score?”); the factor cumulative contribution rate was 66.178% by exploratory factor analysis (EFA). By confirmatory factor analysis (CFA), the chi-square value (χ2) was 662.780; the degrees of freedom (df) was 242; the chi-square value/degrees of freedom (χ2/df) was 2.739; the root-mean-square error of approximation (RMSEA) was 0.049; the goodness of fit index (GFI) was 0.929; the adjusted goodness of fit index (AGFI) was 0.905; the comparative fit index (CFI) was 0.964; the normed fit index (NFI) was 0.944; and the Tueker–Lewis index (TLI) was 0.955. AHRKAAS consists of 4 dimensions and 25 items, namely the cognition of human factors of haze formation (7 items), the cognition of natural factors of haze formation (4 items), the cognition of haze harmful effects on the human body (9 items), and the cognition of haze health protection measures (5 items). AHRKAAS used the Likert 5-point method (5 = completely know; 4 = know most; 3 = moderately know; 2 = know a small part, 1 = don't know). The total score of the scale ranged from 25 to 125 points. The higher the AHRKAAS score, the higher the level of adolescent haze-related knowledge.

### Ethical Consideration and Survey Method

The Health and Family Planning Commission of Hebei province approved this study (Permit Number: 20150072). The study was also approved by the Medical Ethics Committee of Hebei University. The research team explained the purpose of this study to the middle school teaching management departments, parents/guardians, and adolescents in two middle schools. Parental/guardian written informed consent was obtained for the adolescents. As soon as the research team obtained school leaders' consents, parents' consents/guardians' consents, and adolescents' assents, the research team explained to the participants how to fill out the questionnaires. By using the standardized language and unified instructions, the questionnaires were anonymously completed by the adolescents.

### Statistical Methods

Epidata 3.1 software was used to input the data into the computer twice and complete a consistency check. The data for continuous variables were reported as means ± standard deviation (SD) and categorical variables were presented as percentages (%). In this study, the normality of the data was confirmed using a probability–probability plot. Independent-sample *t*-test and analysis of variance (ANOVA) were used for continuous variables. A multivariable linear regression model was used to analyze related factors that could affect the adolescent haze-related knowledge level. The multivariable linear regression model formula was as follows:

$$\begin{array}{l} {\text{Y}}_{\text{AHRKAAS}}=\beta_{0}+\beta_{1}{\text{X}}_{1}+\beta_{2}{\text{X}}_{2}+\beta_{3}{\text{X}}_{3}+\ldots +\beta_{\text{m}}{\text{X}}_{\text{m}}+e \end{array}$$

Y_AHRKAAS_ represented the AHRKAAS overall score that pertained to the level of adolescent haze-related knowledge. The AHRKAAS overall score was the sum of all of the items' scores in the AHRKAAS. X_1…_X_m_ represented different covariates, such as demographics, medical factors, and knowledge of respiratory disease. As part of the sensitivity analysis, different models were established to confirm the associations between monthly expenses, living condition, physical examination frequency, knowledge of respiratory system diseases, and the AHRKAAS score. Regarding the sensitivity analysis, Model 1 adjusted for four influencing factors (monthly expenses, living condition, physical examination frequency, and knowledge of respiratory system diseases) in the multivariable linear regression model. Model 2 adjusted for four influencing factors (monthly expenses, living condition, physical examination frequency, and knowledge of respiratory system diseases) and other demographic characteristics (sex, grade, race, religious faith, place of residence, type of medical insurance, and chronic respiratory disease) in the multivariable linear regression model. The IBM SPSS Statistics 22.0 software (SPSS, Chicago, IL, USA) was used to conduct the statistical analysis. A *P* < 0.05, in two sides, was considered statistically significant.

## Results

### The AHRKAAS Overall Score and Individual Dimension Scores Among the Adolescents

The AHRKAAS score level of adolescents ranged from 25 points to 125 points. Because the different dimensions of AHRKAAS included the different numbers of items, the total score of AHRKAAS and the score of each dimension were transformed by the score rate. The score rate = total score (or score of each dimension) ÷ scale maximum possible score (or each dimension maximum possible score) * 100%. The AHRKAAS overall score of adolescents was 87.314 ± 20.227 (the score rate was 69.9%). In each dimension, the dimension score rate of cognition of human factors of haze formation was the highest (the score rate was 85.6%). The dimension score rate of cognition of the harmful effects of haze on the human body was the lowest (the score rate was 57.1%; Table 1).

**Table 1:** Total score of AHRKAAS and the score of each dimension.

**Dimension**	**Number of items**	**Score (means ± SD)**	**Score rate (%)**
The cognition of human factors of haze formation	7	29.943 ± 6.602	85.6
The cognition of natural factors of haze formation	4	12.512 ± 4.787	62.6
The cognition of haze harmful effects on the human body	9	25.690 ± 10.484	57.1
The cognition of haze health protection measures	5	19.170 ± 5.205	76.7
Total score	25	87.314 ± 20.227	69.9

Score rate = total score (or score of each dimension) ÷ scale maximum possible score (or each dimension maximum possible score) *100%.

### The Five Items With Higher Scores and the Five Items With Lower Scores in the AHRKAAS

The highest score rate in all items of AHRKAAS was 87.3% (Item: “I know that factory emissions can cause haze”). All of the five items with higher scores were included in the dimension of the cognition of human factors of haze formation (Table 2). The lowest score rate in all items of AHRKAAS was 49.7% (Item: “I know haze can cause reproductive dysfunction”). All of the five items with lower scores were included in the dimension of the cognition of haze harmful effects on the human body (Table 2).

**Table 2:** Five items with higher scores and five items with lower scores in the AHRKAAS.

**Number**	**Item**	**Score (means ± SD)**	**Score rate (%)**
**The 5 items with higher scores**			
1	I know that factory emissions can cause haze.	4.365 ± 1.052	87.3
2	I know that burning garbage can cause haze.	4.336 ± 1.099	86.7
3	I know that the automobile exhaust can cause haze.	4.330 ± 1.092	86.6
4	I know that the forest fires can cause haze.	4.270 ± 1.127	85.4
5	I know that burning agricultural straw can cause haze.	4.269 ± 1.130	85.4
**The 5 items with lower scores**			
1	I know haze can cause reproductive dysfunction.	2.485 ± 1.487	49.7
2	I know haze can cause allergic reactions in the body.	2.496 ± 1.524	49.9
3	I know haze can cause metabolic diseases.	2.639 ± 1.500	52.8
4	I know haze can cause dysfunction of the nervous system.	2.640 ± 1.481	52.8
5	I know haze can cause dysfunction of the arteries.	2.777 ± 1.490	55.5

Score rate = total score (or score of each dimension) ÷ scale maximum possible score (or each dimension maximum possible score) *100%.

### The Relations of Demographic Factors, Medical Factors, and Respiratory Disease Knowledge Factors to AHRKAAS Scores

The adolescent detailed demographic data are shown in Table 3. The differences of AHRKAAS scores were statistically significant in groups pertaining to monthly expenses (*F* = 4.026, *P* = 0.018), living conditions (*t* = 5.275, *P* < 0.001), physical examination frequency (*F* = 9.594, *P* < 0.001), and knowledge of respiratory diseases (*F* = 19.711, *P* < 0.001). The differences of AHRKAAS scores were not statistically significant in different groups pertaining to sex (*t* = 0.860, *P* = 0.390), grade (*t* = 0.762, *P* = 0.446), race (*t* = −1.066, *P* = 0.287), place of residence (*t* = 1.194, *P* = 0.233), and type of medical insurance (*F* = 0.574, *P* = 0.564) (Table 3).

**Table 3:** Relations of demographic factors, medical factors, and respiratory disease knowledge factors to AHRKAAS score.

**Demographic data**	***n* (%)**	**Total score**	***t* or F**	***P*-value**
		**(means ± SD)**	**value**	
**Sex^§^**				
Male	519 (50.2)	87.853 ± 20.808	0.860	0.390
Female	515 (49.8)	86.771 ± 19.630		
**Grade^§^**				
Junior high school	713 (69.0)	87.628 ± 20.591	0.762	0.446
Senior high school	321 (31.0)	86.615 ± 19.407		
**Race^§^**				
Han	999 (96.6)	87.188 ± 20.218	−1.066	0.287
Minority	35 (3.4)	90.894 ± 20.453		
**Monthly expenses (yuan)^※^**				
<300	345 (33.4)	86.708 ± 20.199	4.026	0.018^*^
300	541 (52.3)	86.511 ± 20.060		
600	148 (14.3)	91.663 ± 20.490		
**Do you have a religious faith^§^**				
No	960 (92.8)	87.335 ± 19.975	0.108	0.914
Yes	74 (7.2)	87.034 ± 23.401		
**Place of residence^§^**				
Urban area	685 (66.2)	87.850 ± 20.505	1.194	0.233
Rural area	349 (33.8)	86.262 ± 19.657		
**Type of medical insurance^※^**				
Urban medical insurance	599 (57.9)	87.873 ± 20.528	0.574	0.564
New rural cooperative medical system	309 (29.9)	86.391 ± 19.693		
Self-paying	126 (12.2)	86.921 ± 20.146		
**Do you live with your parents^§^**				
Yes	967 (93.5)	88.176 ± 19.906	5.275	<0.001^*^
No	67 (6.5)	74.869 ± 20.878		
**Physical examination frequency^※^**				
Never	86 (8.3)	79.219 ± 18.809	9.594	<0.001^*^
Once a few years	264 (25.5)	84.488 ± 20.314		
Once a year	528 (51.1)	88.870 ± 19.626		
A few times a year	156 (15.1)	91.290 ± 21.183		
**Do you suffer from a long-term chronic respiratory disease^§^**				
No	983 (95.1)	87.170 ± 20.075	−1.008	0.314
Yes	51 (4.9)	90.097 ± 23.000		
**Do your relatives or friends suffer from a long-term chronic respiratory disease^§^**				
No	921 (89.1)	87.068 ± 20.330	−1.115	0.265
Yes	113 (10.9)	89.316 ± 19.339		
**Do you often see patients with a long-term chronic respiratory disease in real life^§^**				
No	907 (87.7)	87.103 ± 20.311	−0.898	0.370
Yes	127 (12.3)	88.823 ± 19.629		
**The understanding degree of knowledge regarding respiratory system diseases^※^**				
Know nothing	38 (3.7)	82.004 ± 25.294	19.711	<0.001^*^
Know a little	148 (14.3)	80.572 ± 19.315		
Know some parts	525 (50.8)	84.983 ± 18.825		
Know most	269 (26.0)	93.936 ± 19.920		
Know very well	54 (5.2)	99.202 ± 20.377		

*Values are means ± SD in cases of continuous variables and numbers (percentages) in case of categorical data*.

§ *Independent-sample t-test was used to analyze the data*.

※ *Analysis of variance (ANOVA) was used to analyze the data*.

* *P < 0.05 was considered to be statistically significant*.

### Multivariable Linear Regression Analysis of Influencing Factors of Adolescent Haze-Related Knowledge Level

The AHRKAAS total score was regarded as the dependent variable. Demographic factors, medical factors, and respiratory disease knowledge factors were regarded as independent variables. The multivariable linear regression model was established. The results showed that four independent variables (monthly expenses, living condition, physical examination frequency, and knowledge of respiratory system diseases) affected the adolescent haze-related knowledge level. Compared with the group (monthly expenses < 300 yuan), the group (monthly expenses ≥ 600 yuan) had a higher haze-related knowledge level (β = 4.882, 95% CI: 0.979, 8.784). Compared with the group (Do not live with parents), the group (Live with parents) had a higher haze-related knowledge level (β = 14.675, 95% CI: 9.494, 19.855). Compared with the group (Never undergo a physical examination), the group (Once a year) (β = 7.444, 95% CI: 2.922, 11.966) and the group (A few times a year) (β = 7.643, 95% CI: 2.367, 12.919) had a higher level of haze-related knowledge. Compared with the group (Know nothing about respiratory system diseases), the group (Know most) (β = 9.623, 95% CI: 2.929, 16.316) and the group (Know very well) (β = 15.367, 95% CI: 7.220, 23.515) had a higher level of haze-related knowledge (Table 4).

**Table 4:** Multivariable linear regression analysis of influencing factors of the level of adolescent haze-related knowledge.

**Variable**	**β (95% CI)**	***t* value**	***P*-value**
**Monthly expenses (yuan)**			
<300	Ref.	−	−
300	0.918 (−1.808, 3.644)	0.661	0.509
600	4.882 (0.979, 8.784)	2.455	0.014^*^
**Do you live with your parents (Yes)**	14.675 (9.494, 19.855)	5.558	<0.001^*^
**Physical examination frequency**			
Never	Ref.	−	−
Once a few years	4.436 (−0.341, 9.212)	1.822	0.069
Once a year	7.444 (2.922, 11.966)	3.230	0.001^*^
A few times a year	7.643 (2.367, 12.919)	2.843	0.005^*^
**The understanding degree of the knowledge about respiratory system diseases**			
Know nothing	Ref.	−	−
Know a little	−3.030 (−10.013, 3.953)	−0.851	0.395
Know a small part	1.041 (−5.450, 7.531)	0.315	0.753
Know most	9.623 (2.929, 16.316)	2.821	0.005^*^
Know very well	15.367 (7.220, 23.515)	3.701	<0.001^*^
**Sex (Female)**	0.696 (−1.706, 3.097)	0.569	0.570
**Grade (Senior high school)**	3.745 (−0.087, 7.577)	1.918	0.055
**Race (Minority)**	3.245 (−3.322, 9.811)	0.970	0.332
**Do you have a religious faith (Yes)**	−0.985 (−5.662, 3.692)	−0.413	0.680
**Place of residence (Rural area)**	−1.215 (−5.044, 2.614)	−0.623	0.534
**Type of medical insurance**
Urban medical insurance	Ref.	−	−
New rural cooperative medical system	−0.391 (−3.530, 2.748)	−0.244	0.807
Self-paying	−0.985 (−4.748, 2.779)	−0.513	0.608
**Do you suffer from a chronic respiratory disease (Yes)**	0.002 (−5.812, 5.815)	0.001	0.9996
**Do your relatives or friends suffer from a chronic respiratory disease (Yes)**	2.485 (−1.630, 6.600)	1.185	0.236
**Do you often see patients with a chronic respiratory disease in real life (Yes)**	1.268 (−2.617, 5.153)	0.641	0.522

*The exploration of influencing factors was conducted by using multivariable linear regression model*.

* *P < 0.05 was considered to be statistically significant*.

### Sensitivity Analysis of the Associations Between Monthly Expenses, Living Condition, Physical Examination Frequency, Knowledge of Respiratory System Diseases, and AHRKAAS Scores

In model 1, four influencing factors (monthly expenses, living condition, physical examination frequency, and knowledge of respiratory system diseases) were included in the multivariable linear regression model. Compared with the group (monthly expenses < 300 yuan), the group (monthly expenses ≥ 600 yuan) also had a higher level of haze-related knowledge (β = 6.035, 95% CI: 2.333, 9.736). Compared with the group (Do not live with parents), the group (Live with parents) also had a higher level of haze-related knowledge (β = 12.542, 95% CI: 7.699, 17.384). Compared with the group (Never undergo a medical examination), the group (Once a year) (β = 7.289, 95% CI: 2.815, 11.762) and the group (A few times a year) (β = 7.095, 95% CI: 1.887, 12.304) also had a higher level of haze-related knowledge. Compared with the group (Know nothing about respiratory system diseases), the group (Know most) (β = 10.301, 95% CI: 3.666, 16.936) and the group (Know very well) (β = 15.666, 95% CI: 7.601, 23.732) also had a higher level of haze-related knowledge.

**Figure 2:**
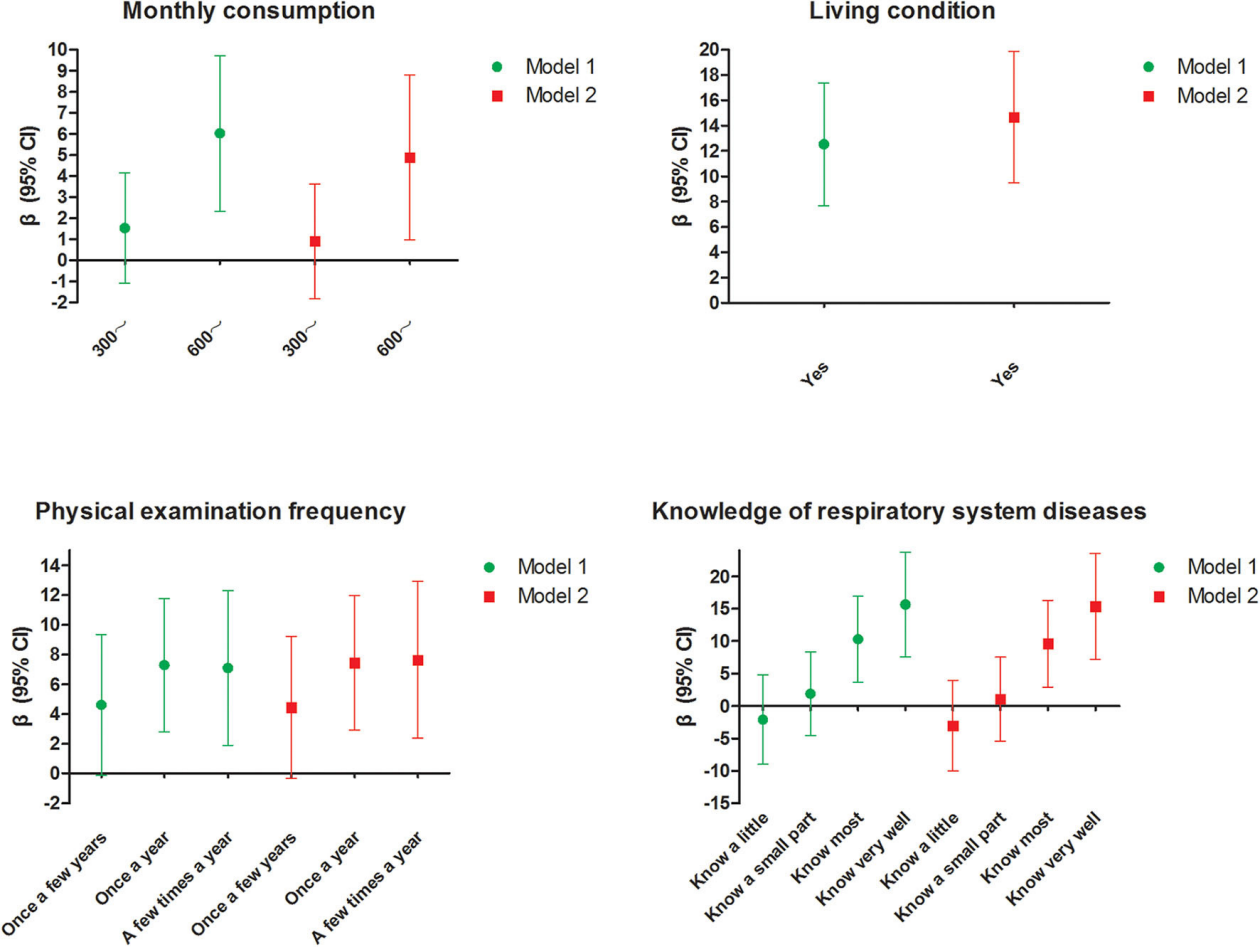
Sensitivity analysis of the associations between monthly expenses, living condition, physical examination frequency, knowledge of respiratory system diseases, and the AHRKAAS score in different models. Model 1 adjusted for four influencing factors (monthly expenses, living condition, physical examination frequency, and knowledge of respiratory system diseases) in the multivariable linear regression model. Model 2 adjusted for four influencing factors (monthly expenses, living condition, physical examination frequency, and knowledge of respiratory system diseases) and other demographic characteristics (sex, grade, race, religious faith, place of residence, type of medical insurance, and chronic respiratory disease) in the multivariable linear regression model.

In model 2, after adjusting for other factors (sex, grade, race, religious faith, place of residence, type of medical insurance, and chronic respiratory disease), four influencing factors (monthly expenses, living condition, physical examination frequency, and knowledge of respiratory system diseases) were still significant and reliable. The detailed results are shown in Table 5. Thus, the results of sensitivity analysis in different models were consistent and reliable (Table 5 and Figure 2).

**Table 5:** Sensitivity analysis of the associations between monthly expenses, living condition, physical examination frequency, knowledge of respiratory system diseases, and the AHRKAAS score in different models.

**Variable**	**Model 1**			**Model 2**		
	**β (95% CI)**	***t*-value**	***P*-value**	**β (95% CI)**	***t*-value**	***P*-value**
**Monthly expenses (yuan)**						
<300	Ref.	−	−	Ref.	−	−
300	1.539 (−1.087, 4.164)	1.150	0.250	0.918 (−1.808, 3.644)	0.661	0.509
600	6.035 (2.333, 9.736)	3.199	0.001^*^	4.882 (0.979, 8.784)	2.455	0.014^*^
**Do you live with your parents (Yes)**	12.542 (7.699, 17.384)	5.082	<0.001^*^	14.675 (9.494, 19.855)	5.558	<0.001^*^
**Physical examination frequency**						
Never	Ref.	−	−	Ref.	−	−
Once a few years	4.610 (−0.136, 9.355)	1.906	0.057	4.436 (−0.341, 9.212)	1.822	0.069
Once a year	7.289 (2.815, 11.762)	3.197	0.001^*^	7.444 (2.922, 11.966)	3.230	0.001^*^
A few times a year	7.095 (1.887, 12.304)	2.673	0.008^*^	7.643 (2.367, 12.919)	2.843	0.005^*^
**The understanding degree of the knowledge about respiratory system diseases**						
Know nothing	Ref.	−	−	Ref.	−	−
Know a little	−2.111 (−8.997, 4.775)	−0.602	0.548	−3.030 (−10.013, 3.953)	−0.851	0.395
Know a small part	1.893 (−4.520, 8.307)	0.579	0.563	1.041 (−5.450, 7.531)	0.315	0.753
Know most	10.301 (3.666, 16.936)	3.047	0.002^*^	9.623 (2.929, 16.316)	2.821	0.005^*^
Know very well	15.666 (7.601, 23.732)	3.812	<0.001^*^	15.367 (7.220, 23.515)	3.701	<0.001^*^

*The exploration of influencing factors was conducted by using a multivariable linear regression model*.

*Model 1 adjusted for four influencing factors (monthly expenses, living condition, physical examination frequency, and knowledge of respiratory system diseases) in the multivariable linear regression model*.

*Model 2 adjusted for four influencing factors (monthly expenses, living condition, physical examination frequency, and knowledge of respiratory system diseases) and other demographic characteristics (sex, grade, race, religious faith, place of residence, type of medical insurance, and chronic respiratory disease) in the multivariable linear regression model*.

* *P < 0.05 was considered to be statistically significant*.

## Discussion

At present, the health care model has gradually transformed from the old model into the new model. In the old model, the medical workers have a leading role in the patient's health care. However, in the new modern model, patients are placed at the center of their health care (18, 19). Therefore, by fully mobilizing adolescent enthusiasm regarding health protection against haze, we can encourage them to adopt positive health protection behaviors in times of hazy weather. This can effectively reduce the haze damages for adolescents. Retrospective studies have demonstrated that many physical symptoms are exacerbated or worsened by haze. The physical symptoms include dry mouth or sore throat, eye discomfort, nose discomfort, shortness of breath, headache, skin irritation, and so on (20, 21). Kelishadi pointed out that air pollution was an important environmental risk factor for pre-hypertension, and exposure to particulate matters in haze might raise blood pressure within hours or days (22). Emmanuel reported that it was more common to see cases of serious respiratory conditions, such as bronchitis and emphysema, in periods of haze exposure (23). Many studies arising from countries affected by serious haze revealed consistent deleterious effects of haze on the respiratory system, cardiovascular system, neurological system, as well as psychological health (21). Given the haze-serious harmful effects on health (12, 24–30), the health protection of haze should be considered among the adolescents. According to knowledge-attitude-practice theory (31), we should help adolescents obtain enough haze-related knowledge, help them adopt correct health protection habits, and finally encourage them to take effective health protection measures. This will reduce the incidence of haze-related diseases among the adolescents, promote the normal growth and development of adolescents, and ultimately result in a reduction of the medical burden placed on families, schools, and government bodies (32).

Table 1 shows that the AHRKAAS overall score of adolescents was 87.314 ± 20.227 (score rate = 69.9%). This showed that the overall cognitive level of haze-related knowledge among the adolescents was relatively low and their haze-related knowledge level needed to be improved. In the score rates of all dimensions, the dimension score rate of the cognition of human factors of haze formation was the highest. This showed that, in recent years, due to the media publicity of this issue, the spread of network information, and the revision and improvement of Chinese laws and policies related to air pollution control measures, adolescents already seemed to have some level of knowledge about human causes of haze formation (for example, factory emissions, burning garbage, automobile exhaust, and so on). All five items with higher scores were also included in the dimension of knowledge of human factors of haze formation (Table 2). The dimension score rate of the cognition of haze harmful effects on the human body was the lowest. This showed that the adolescents did not have sufficient basic knowledge about haze-related harms on the human body. All five items with lower scores were also included in the dimension of the cognition of haze harmful effects on the human body (Table 2). At present, a great deal of research regarding haze occupies the basic research field, for example, the harmful effects of haze on physiological functions, and its haze-damaging effects on tissues and organs (13, 22, 33). These research achievements help to study the occurrence and medical treatment of haze-related diseases. However, these basic research achievements have not been widely applied to the haze health protection field among adolescents. In Chinese middle schools, due to lack of health care classes or lectures about this issue, it is difficult for adolescents to obtain enough knowledge about haze-damaging effect on the body. Therefore, in the future, more publicity and education programs of haze need to be implemented. It is important to teach adolescents the haze-specific impacts on body health and better encourage their willingness to adopt protective health behaviors.

Tables 3, 4 show that the level of adolescent haze-related knowledge was affected by monthly expenses, living condition, physical examination frequency, and knowledge of respiratory system diseases. Table 5 and Figure 2 showed that the effects of four influencing factors on adolescent haze-related knowledge were reliable and consistent in different sensitivity analysis models. The haze-related knowledge level of adolescents who live with their parents was higher. Compared with adolescents with lower monthly expenses (<300 yuan), the haze-related knowledge level of adolescents with higher monthly expenses (≥600 yuan) was also higher. Due to the physiological and psychological immaturity of adolescents, the knowledge that adolescents possessed regarding prevention, to a very great extent, is proportionate to the level of teaching obtained by their parents. Moreover, the amount of adolescent monthly expenses, to a certain extent, reflects the economic level of their families. Wang et al. (31) pointed out that parents' level of education as well as the family income status were two important factors that affected a young person's ability to protect himself or herself from air pollution. On the other hand, parents with a higher education level and a higher economic income level also had better awareness of how to protect themselves. Kicinski found that the education level of the adolescents' mothers was an important factor that affected the association between the air pollution exposure and the neurobehavioral performance among the adolescents (34). Therefore, this indicates that we should not only strengthen haze health protection education for adolescents but also provide relevant health guidance for their parents in the future in order to achieve the best effect of haze health protection. According to the Health Belief Model (35), adolescents with higher physical examination frequency usually have a better health protection awareness and realize the importance of health care; moreover, the physical examination center is also a very good health education platform and provides a variety of health care information. Therefore, this indicates that physical examination platforms may play an important role in the process of publicity, education, and behavioral intervention for adolescent haze health protection. The adolescent who has a better understanding of knowledge about respiratory system diseases also has a higher haze-related knowledge level. Haze is an important risk factor that leads to many respiratory system diseases (14, 15, 36). Haze can directly enter the body through the respiratory system. These harmful particle attaches to the respiratory tract mucous membrane, deposits in the alveoli, damages the integrity of the alveolar epithelial cells, reduces the lung capacity, and causes consequential respiratory-related diseases such as rhinitis, bronchitis, pneumonia, and lung cancer (15, 36). The more the adolescents pay attention to information regarding respiratory system diseases, the greater their chances of obtaining haze-related knowledge. Therefore, this indicates that, in the future, during phases of prevention and treatment of adolescent respiratory system diseases, medical institutions should emphasize the importance of haze-related education. Our study is not free from limitations, however; physical symptoms including dry mouth, sore throat, eye discomfort, nose discomfort, shortness of breath, headache, and skin irritation were not included in the scale (AHRKAAS). In the future, researchers will add the contents of these physical symptoms to further revise and improve the scale. In the future, researchers will add more items and more contents to further revise and improve the AHRKAAS.

## Conclusion

The overall cognitive level of adolescent haze-related knowledge is relatively low and is related to monthly expenses, living condition, physical examination frequency, and knowledge of respiratory system diseases. Government, society, and individuals should take more measures to reduce air pollution. Government bodies, schools, and research institutions should strengthen cooperation to identify preventable risk factors, and take positive intervention measures to reduce harmful impacts of haze on adolescent health. Health care publicity and health education activities regarding haze-related knowledge among adolescents are needed to improve the adolescents' capability to protect themselves from the effect of haze. Ultimately, these effective measures can promote the healthy growth of adolescents.

## Data Availability Statement

The datasets generated for this study are available on request to the corresponding author.

## Ethics Statement

The studies involving human participants were reviewed and approved by the Health and Family Planning Commission of Hebei Province approved this study (Permit Number: 20150072). The study was also approved by Medical Ethics Committee of Hebei University. Written informed consent to participate in this study was provided by the participants' legal guardian/next of kin.

## Author Contributions

QZ and LT conceived and designed the study. YZ, HD, YL, and YC collected and analyzed the data. QZ, YZ, and LT wrote the manuscript. All authors read and approved the final manuscript.

## Conflict of Interest

The authors declare that the research was conducted in the absence of any commercial or financial relationships that could be construed as a potential conflict of interest.

## Abbreviations

AHRKAAS: Adolescent Haze-Related Knowledge Awareness Assessment Scale
RMSEA: Root-mean-square error of approximation
GFI: Goodness of fit index
AGFI: Adjusted goodness of fit index
CFI: Comparative fit index
NFI: Normed fit index
TLI: Tueker–Lewis index
EFA: Exploratory factor analysis
CFA: Confirmatory factor analysis.
