# Supplementary material for: Adolescent Haze-Related Knowledge Level Study: A Cross-Sectional Survey With Sensitivity Analysis
Source: Front Public Health. 2020 Jul 9;8:229. doi: 10.3389/fpubh.2020.00229 (PMC7363765; doi:10.3389/fpubh.2020.00229)
Supplement: Supplementary file 1 [file Table_1.DOCX]

Additional file 1 The content of the Adolescent Haze-related Knowledge Awareness Assessment Scale (AHRKAAS).

| **Dimensions** | **Items** | **Completely know**  **5** | **Know most**  **4** | **Moderately know**  **3** | **Know a small part**  **2** | **Don't know**  **1** |
| --- | --- | --- | --- | --- | --- | --- |
| The cognition of human factors of haze formation | Q1. I know that factory emissions can cause haze. | 5 | 4 | 3 | 2 | 1 |
|  | Q2. I know that burning agricultural straw can cause haze. | 5 | 4 | 3 | 2 | 1 |
|  | Q3. I know that forest fires can cause haze. | 5 | 4 | 3 | 2 | 1 |
|  | Q4. I know that burning garbage can cause haze. | 5 | 4 | 3 | 2 | 1 |
|  | Q5. I know that coal-fired heating can cause haze. | 5 | 4 | 3 | 2 | 1 |
|  | Q6. I know that dust produced from cars can cause haze. | 5 | 4 | 3 | 2 | 1 |
|  | Q7. I know that automobile exhaust can cause haze. | 5 | 4 | 3 | 2 | 1 |
| The cognition of natural factors of haze formation | Q8. I know that when there is no wind, haze can be caused. | 5 | 4 | 3 | 2 | 1 |
|  | Q9. I know that a decrease in rainfall can cause haze. | 5 | 4 | 3 | 2 | 1 |
|  | Q10. I know that when the relative humidity of the air is high, haze can be caused. | 5 | 4 | 3 | 2 | 1 |
|  | Q11. I know that when the temperature is low, haze can be caused. | 5 | 4 | 3 | 2 | 1 |
| The cognition of haze harmful effects on the human body | Q12. I know haze can cause pneumonia. | 5 | 4 | 3 | 2 | 1 |
|  | Q13. I know that haze can cause lung cancer. | 5 | 4 | 3 | 2 | 1 |
|  | Q14. I know that haze can cause the blood pressure to rise. | 5 | 4 | 3 | 2 | 1 |
|  | Q15. I know that haze can cause heart disease. | 5 | 4 | 3 | 2 | 1 |
|  | Q16. I know haze can cause dysfunction of the arteries. | 5 | 4 | 3 | 2 | 1 |
|  | Q17. I know haze can cause dysfunction of the nervous system. | 5 | 4 | 3 | 2 | 1 |
|  | Q18. I know haze can cause metabolic diseases. | 5 | 4 | 3 | 2 | 1 |
|  | Q19. I know haze can cause reproductive dysfunction. | 5 | 4 | 3 | 2 | 1 |
|  | Q20. I know the haze can cause allergic reactions in the body. | 5 | 4 | 3 | 2 | 1 |
| The cognition of haze health protection measures | Q21. I know that window opening time should be reduced in haze. | 5 | 4 | 3 | 2 | 1 |
|  | Q22. I know that outdoor activities should be reduced in haze. | 5 | 4 | 3 | 2 | 1 |
|  | Q23. I know that I should wear a protective mask in haze. | 5 | 4 | 3 | 2 | 1 |
|  | Q24. I know that I should relax my mood in haze. | 5 | 4 | 3 | 2 | 1 |
|  | Q25. I know that I should maintain enough sleep in haze. | 5 | 4 | 3 | 2 | 1 |
